# LncRNAs act as prognostic biomarkers in bladder carcinoma: A meta-analysis

**DOI:** 10.1016/j.heliyon.2019.e02785

**Published:** 2019-11-14

**Authors:** Yucheng Zhong, Yeshen Zhang, Huan Li, Wenmin Ma

**Affiliations:** a Assisted Reproductive Technology Center, Foshan Maternity and Children's Healthcare Hospital Affiliated to Southern Medical University, Foshan, 528000, China; b School of Clinical Medicine, Guangdong Pharmaceutical University, Guangzhou, 510006, China

**Keywords:** Cell biology, Molecular biology, Cancer research, Urology, Oncology, LncRNA, Prognosis, Bladder carcinoma, Meta-analysis

## Abstract

**Background and purpose:**

Increasing studies have shown that different kinds of lncRNAs play key role in the development of multiple carcinomas. Therefore, we conducted a meta-analysis to investigate an association between the expression level of lncRNAs and the prognosis of bladder cancer (death or other clinical outcomes).

**Methods:**

A systematic literature search was performed by using PubMed. Twenty-four studies were included in the meta-analysis based on the inclusion and exclusion criteria. In total, there are 1652 independent participants.

**Results:**

The result showed that high expression levels of lncRNAs were demonstrated to be associated with poor overall survival (OS) (HR = 2.33, 95%CI: 1.51–2.39, *p* < 0.01) in bladder carcinoma, but there was no significant correlation between lncRNAs level and recurrence-free survival (RFS) (pooled HR = 1.57, 95%CI 0.69–3.56, *p* = 0.284), and progression-free survival (PFS) (pooled HR = 1.37, 95%CI 0.79–2.38, *p* = 0.269). Additionally, increased lncRNAs expression was found to be moderately correlated with tumor stage and progression (II/III/IV vs. I, OR = 3.20, 95%CI: 1.72–5.98, *p* < 0.001). In addition, elevated lncRNAs expression predicted lymph node metastasis (LNM) significantly (pooled OR = 2.29, 95 % CI 1.33–3.95, *p* < 0.01). No significant heterogeneity was observed among studies except lymph node metastasis.

**Conclusion:**

In conclusion, high expression levels of lncRNAs were demonstrated to be associated with poor OS and positive LNM, and lncRNAs might be potential prognostic markers in bladder cancer.

## 1 Introduction

With the increasing incidence and mortality of cancer in China, it has become a major cause of death and public health problem all over the word. It is estimated that 4292,000 new cancer cases and 2814,000 cancer deaths would occur in China in 2015 [1]. According to the recent study, bladder cancer is one of the most common malignant tumors in the world and the most common urologic tumors in China [2]. During the past decade, the incidence and mortality of bladder cancer have been notably increased [3]. However, there are no specific symptoms for these patients who are at the early stage of bladder cancer, and most of the patients are at an advanced stage when they go to the hospital at the first time [4]. Surgery is known to be the primary treatment for bladder cancer, but recurrence and metastasis are still common. Since the prognosis of bladder cancer is closely related to the stage of disease at diagnosis, it is urgently needed to find out markers that more sensitive and specific for diagnosis at early stages [5].

LncRNAs are a class of noncoding RNAs which are greater than 200 nucleotides in length and have the limited coding potential [6]. Along with the rapid development of the whole genome analysis technology, a growing body of evidence indicates that lncRNAs play a role in a serious of cellular processes, including cell growth, survival, migration, and differentiation. Besides, lncRNAs, as an important tumor regulator, has been widely concerned due to its potential role in tumor development, progression, and metastasis, such as TUG-1, UCA-1, MALAT1 and so on [7]. Many researchers have found that lncRNAs regulate gene expression and pathophysiological processes at the level of transcription, post-transcriptional, and epigenetic through histone modifications, transcriptional interference, imprinting, chromatin remodeling, cell cycle control, and selective splicing. Recently, more and more studies have suggested that lncRNAs, such as UCA-1, MALAT1, PANDAR and so on, play key roles in development and progression of bladder cancer. UCA1 is the first lncRNA that acknowledged in human bladder cancer [8]. Here, we maded this meta-analysis to discover the association between expression level of different lncRNAs and prognosis of the patients with bladder tumor.

## 2 Materials and methods

### 2.1 Meta analysis

This report is strictly in accordance with the PRISMA guidelines [9]. All analyses were based on previously published studies, thus there is no need for ethical approval and patient consent.

### 2.2 Search strategy

Comprehensive literature retrieval was performed on PubMed. The literature search was conducted up to Sep. 20, 2018. The publications were identified with the combination of the following search terms: ((((((((((((((Noncoding RNA, Long[Title/Abstract]) OR lncRNA[Title/Abstract]) OR Long ncRNA[Title/Abstract]) OR ncRNA, Long[Title/Abstract]) OR Long Non-Coding RNA[Title/Abstract]) OR RNA, Long Non-Coding[Title/Abstract]) OR Long ncRNAs[Title/Abstract]) OR ncRNAs, Long[Title/Abstract]) OR LincRNAs[Title/Abstract]) OR LINC RNA[Title/Abstract])) OR ""RNA, Long Noncoding""[Mesh])) AND ((""Urinary Bladder""[Mesh]) OR ((Bladder, Urinary[Title/Abstract]) OR Bladder[Title/Abstract]))) AND ((""Neoplasms""[Mesh]) OR (((((((((((((Neoplasia[Title/Abstract]) OR Neoplasias[Title/Abstract]) OR Neoplasm[Title/Abstract]) OR Tumors[Title/Abstract]) OR Tumor[Title/Abstract]) OR Benign Neoplasms[Title/Abstract]) OR Neoplasms, Benign[Title/Abstract]) OR Benign Neoplasm[Title/Abstract]) OR Neoplasm, Benign[Title/Abstract]) OR Malignancy[Title/Abstract]) OR Malignancies[Title/Abstract]) OR Cancer[Title/Abstract]) OR Cancers[Title/Abstract])). In order to avoid possible omissions, we also carefully scanned the references of relevant reviews and research articles. Firstly, we excluded duplicate articles. Secondly, we scanned the title and summary. Thirdly, the full text of possible qualified studies were carefully reviewed. The retrieved literature was examined in detail to rule out potential duplications. This study is based on the PRISMA statement for prediction, implementation and reporting.

### 2.3 Inclusion and exclusion criteria

A study was included if it met the following criterions: (1) The study should be investigated in the association between lncRNAs with bladder cancer patients. (2) Cancer patients were divided into two levels, high or low, on the basis of the expression levels of lncRNAs which were measured in primary tumor tissues. (3) The study investigated the prognostic value of patients with survival outcomes, such as OS/chemical recurrence-free survival (BCR-FS)/recurrence-free survival (RFS)/disease-free survival (DFS)/metastasis-free survival (MFS)/cancer-specific survival (CSS)/progression-free survival (PFS)), and provided a hazard ratios (HR) or relative risk (RR), 95% CI or p -value, and Kaplan-Meier curves or required data obtained by contacting corresponding authors. (4) Eligible studies should contain clinical pathological characteristics like tumor state of cancers (T), lymph node metastasis (LNM), or distant metastasis (DM). (5) The full-text paper was available.

On the other hand, a study was excluded based on the criteria below: (1) Duplicate publications. (2) Nonhuman study or non-clinical study. (3) Basic research or Animal experiments. (4) non-English paper or no full text. (5) Reviews, case reports, letters, editorials, and expert opinions. (6) Studies were not grouped according to the expression level of lncRNAs. (7) Studies without available data.

### 2.4 Quality assessment

Two investigators (Y. Zhong and Y. Zhang) independently assessed the quality of all the included diagnostic studies using the NEW CASTLE-OTTAWA QUALITY ASSESSMENT SCALE (NOS). NOS was divided into three parts including selection, comparability, and outcome, which evaluated the quality of the articles objectively and comprehensively. The scores of NOS criteria were ranged from 0 (lowest) to 8 (highest). If the final scores of a study were higher, the methodological quality was better. A study with a NOS score equal or more than 5 was considered to be of high quality. In this meta-analysis, the quality of all studies included in this meta-analysis was varied from 5 to 8, with a mean value of 5.8.

### 2.5 Data extraction

Eligible articles were reviewed independently by two investigators (H. Li and W. Ma), with which disagreements were resolved by discussion. We abstracted the following information from each study: (1) Publication information: including first author; year of publication; country of origin; (2) patients’ characteristic information: type of lncRNAs, clinical tumor stage, number of participants and follow-up duration; (3) lncRNAs information: tissue sample, detection method of the lncRNAs, cut-off values expression associates with poor prognosis and number of high lncRNAs expression group and low lncRNAs expression group; (4) Prognosis information: including the relationship between lncRNAs level and the number of patients with lymph nodes metastasis, distant metastasis, different tumor state, tumor grade; (5) Survival analysis and multivariate analysis, containing HR and corresponding 95% Cl is for OS, RFS, DFS, PFS and CSS. If available, these data were obtained from the original article; otherwise, contacting the corresponding author to collect these data; if Kaplan-Meier curves were available, data were extracted from graphical survival plots and HRs were estimated.

### 2.6 Statistical methods

All analyses were performed using the STATA software version 14.0. To investigate the heterogeneity among studies, I2 statistics, and chi-square Q test was used. When I2 value more than 50% or a p-value less than 0.05 for Q test, the heterogeneity was regarded as significant. Random-effects model was used whether there was significant heterogeneity between studies or not. A “forest plots” was used to show the content of this statistical analysis.

The HRs and 95% CI were used to evaluate the association between lncRNAs and prognosis and LNM. On one hand, a provided HR > 1 meant a poor survival or more susceptibility to develop LNM for the high expressed lncRNAs group. On the other hand, HR < 1 indicated a worse survival or more susceptibility to develop LNM for the group with decreased lncRNAs expression level. We extracted HR according to the following two methods: (1) The HRs and 95% CI were obtained directly from the publication; (2) We calculated the HRs and 95%CI by extracting several survival rates from the Kaplan-Meier survival curves using Engauge Digitizer version 4.1 (free software downloaded from http://sourceforge.net). The second method may generate errors by variation. Meanwhile, the ORs and 95% CI were used for investigating the relationship between the expression of lncRNAs and clinicopathologic characteristics. Sensitivity analysis was also performed to test the effect of each study on the pooled results. The Begg's test was used to assess publication bias. When p < 0.05 was considered to be statistically significance.

## 3 Results

### 3.1 Characteristics and eligible studies

**Fig. 1:**
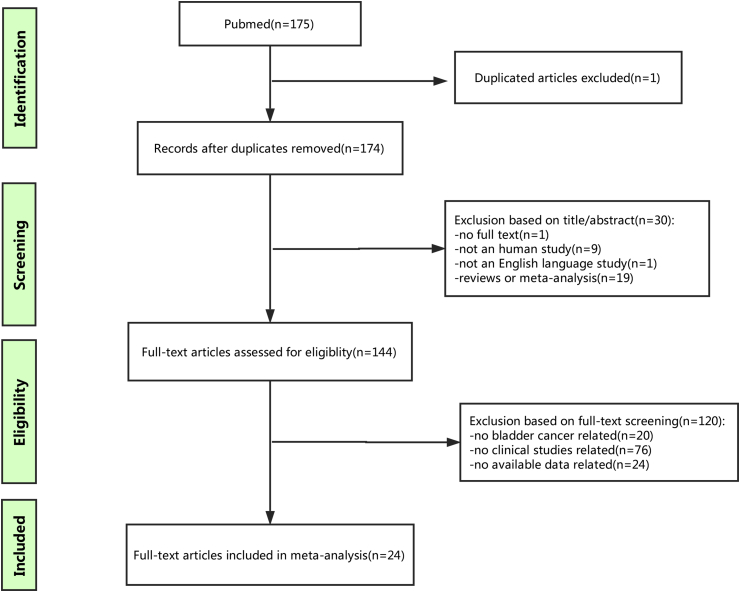
The flow diagram indicated the process of study selection.

Initially, 175 publications were found through the internet search from PubMed. One duplicated article was excluded. After a detailed screening of the title and abstract, 30 records were excluded for the following reasons: one with no full text, one not an English study, eight not human studies, nineteen reviews or meta-analysis. Then, after further evaluation of the full text, 120 studies, including twenty irrelevant with bladder cancer, 76 irrelevant with clinical studies, 24 without available data, were further excluded on the basis of the exclusion criteria. As a result, a total of 24 articles met the inclusion criteria and were included in the final analysis. All of the selected studies were non-randomized. A flow diagram of the study selection process is shown in Fig. 1.

All of the studies were published recently (2013–2018). These studies included a total of 1652 patients. Fifteen different types of lncRNAs were evaluated in this meta-analysis: SPRY4-IT1, SUMO1P3, NEAT1, n336928, MIR31HG, BANCR, HOXD-AS1, HOTAIR, PANDAR, UNMIBC, PVT1, TINCR, HIF1A-AS2, AK127730, AK130230, ABO74278, AF075063, BC01507, AK122774, CCERP, ABHD11-AS1, AATBC, GHET1, CCAT2, TUG1, MALAT1, GAS5, UBC1 [10, 11, 12, 13, 14, 15, 16, 17, 18, 19, 20, 21, 22, 23, 24, 25, 26, 27, 28, 29, 30, 31, 32]. More than 80% of the studies were from China. All the detected samples were tissues or frozen tissues from the patients before any anti-cancer treatment. The quantitative reverse transcription polymerase chain reaction (qRT-PCR) method was used to measure the expression of lncRNAs in all these studies. Cut-off scores that discriminate high and low lncRNAs expression were selected by ROC curve or median value or X-tile algorithm or fold change level while two studies did not mention. There were six studies for OS [10, 12, 17, 19, 27, 30], two for RFS [19, 31], one for PFS [23], one for both OS and DFS [32], one for RFS and PFS [16] enrolled in the database-based analysis. All of the diagnoses of lymph node metastasis were based on pathology. The Newcastle-Ottawa Scale (NOS) was used to confirm that all the studies were of good quality. The main characteristics of the included articles were summarized in Table 1. The quality assessment of eligible studies was showed in Table 2.

**Table 1:** Characteristics of studies in this meta-analysis.

Study	Year	Country	LncRNA	Total number	Detection method	Cut-off	lncRNA expression						Survival analysis	Multivariate analysis	HR statistic	Hazard ratios (95% CI)	Follow-up, moths
							High expression	Low expression	High with T2-4/3-4	Low with T2-4/3-4	High with LNM	Low with LNM					
Zhao	2015	China	SPRY4-IT1	68	qRT-PCR	Mean	38	30	25	11	18	1	OS	Yes	Rep	3.72 (2.08–6.72)	60 Total
Zhan	2016	China	SUMO1P3	55	qRT-PCR	X-tile algorithm	38	17	15	2	1	1	NA	Unreported			NA
Chen	2016	China	NEAT1	65	qRT-PCR	X-tile algorithm	48	17	33	2	3	0	NA	Unreported			NA
Chen	2015	China	n336928	95	qRT-PCR	Median	44	51	35	15			OS	Yes	Rep	2.38 (1.01–5.61)	60 Total
He	2016	China	MIR31HG	55	qRT-PCR	X-tile algorithm	19	36	8	28	3	1	NA	Unreported			NA
He*	2016	China	BANCR	54	qRT-PCR	X-tile algorithm	19	35	9	30	1	3	NA	Unreported			NA
Li	2016	China	HOXD-AS1	50	qRT-PCR	X-tile algorithm	30	20	15	6	5	1	NA	Unreported			NA
Fernández	2015	Spain	HOTAIR	66	qRT-PCR	Median	30	33					RFS	Yes	SC	1.02 (0.54–1.93)	40 Total
			HOTAIR	33	qRT-PCR	Median	17	16					PFS	Yes	SC	1.64 (0.50–5.41)	33.3 Total
Yan	2014	China	HOTAIR	110	qRT-PCR	Mean	90	20	0	0			OS	Yes	Rep	4.71 (2.89–8.71)	39 Median
Zhan*	2016	China	PANDAR	55	qRT-PCR	NA	37	18	15	2	1	1	NA	Unreported			NA
Zhang	2016	China	UNMIBC	75	qRT-PCR	NA	42	33	0	0			RFS	Yes	Rep	2.36 (1.50–4.84)	42 Total
Zhuang	2015	China	PVT1	32	qRT-PCR	X-tile algorithm	20	12	19	5	1	2	NA	Unreported			NA
Chen*	2016	China	TINCR	49	qRT-PCR	X-tile algorithm	33	16	25	8	2	0	NA	Unreported			NA
Chen**	2016	China	HIF1A-AS2	44	qRT-PCR	X-tile algorithm	30	14	22	1	3	0	NA	Unreported			NA
Peter	2014	UK	AK127730	56	qRT-PCR	Median	28	28					PFS	Unreported	SC	3.67 (1.12–11.98)	110 Total
			AK130230				27	29								2.17 (0.67–6.66)	
			ABO74278				28	28								1.90 (0.64–5.66)	
			AF075063				27	29								0.68 (0.19–2.32)	
			BC01507				27	29								0.36 (0.05–2.43)	
			AK122774				27	29								0.63 (0.19–2.08)	
Zhan	2017	China	CCERP	55	qRT-PCR	X-tile algorithm	38	17	32	9			NA	Unreported			NA
Chen	2017	China	ABHD11-AS1	66	qRT-PCR	X-tile algorithm	47	19	35	5	6	1	NA	Unreported			NA
Zhao	2014	China	AATBC	90	qRT-PCR	Median	54	36	35	14	12	5	NA	Unreported			NA
Li	2014	China	GHET1	80	qRT-PCR	Median	39	41					OS	Unreported		1.66 (0.38–7.26)	60 Total
Li*	2016	China	CCAT2	48	qRT-PCR	X-tile algorithm	28	20	25	11	1	2	NA	Unreported			NA
Iliev	2016	Czech Republic	TUG1	47	qRT-PCR	ROC curve	26	21					OS	Yes		1.14 (0.43–3.05)	30 Median
Li	2017	China	MALAT1	120	qRT-PCR	Mean	64	56	44	24	21	7	OS	Yes	Rep	2.06 (1.24–3.88)	61.8 Median
Zhang*	2016	China	GAS5	82	qRT-PCR	T/N ratio<*	41	41					RFS	Yes	Rep	0.48 (0.29–0.81)	60 Total
He	2013	China	UBC1	102	qRT-PCR	Mean	60	42	39	24	19	5	OS,	Unreported		1.08 (0.44–2.69)	80 Total
													DFS			1.74 (0.74–3.93)	

OS = overall survival; RFS = recurrence free survival; PFS = progression free survival; DFS = disease-free survival.

**Table 2:** Quality assessment of eligible studies (Newcastle-Ottawa Scale).

Study	Selection			Comparability			Outcome		Total
	Adequacy of case definition	Number of case	Representativeness of the cases	Ascertainment of exposure	Ascertainment of detection method	Ascertainment of cut-off	Assessment of outcome	Adequate follow up	
Zhao2015	1	1	1	1	1	1	1	1	8
Zhan2016	0	1	1	1	1	1	0	0	5
Chen2016	0	1	1	1	1	1	0	0	5
Chen2015	1	1	1	1	1	1	1	1	8
He 2016	0	1	1	1	1	1	0	0	5
He 2016	1	1	1	1	1	1	0	0	6
Li2016	1	0	1	1	1	1	0	0	5
Fernández2015	0	1	1	1	1	1	1	1	7
Yan2014	1	1	1	1	1	0	0	0	5
Zhan2016	1	1	1	1	1	0	1	1	7
Zhang 2016	1	1	1	1	1	0	1	1	7
Zhuang2015	1	0	1	1	1	1	0	0	5
Chen2016	1	0	1	1	1	1	0	0	5
Chen2016	1	0	1	1	1	1	0	0	5
Peter2014	1	1	1	1	1	1	1	0	8
Zhan2017	0	1	1	1	1	1	0	0	5
Chen2017	1	1	1	1	1	1	0	0	6
Zhao2014	1	1	1	1	1	0	0	0	5
Li2014	1	1	1	1	1	1	1	1	8
Li2016	1	0	1	1	1	1	0	0	5
Iliev2016	1	0	1	1	1	1	1	0	6
Li2017	1	1	1	1	1	1	1	1	8
Zhang2016	1	1	1	1	1	1	1	1	8
He2013	1	1	1	1	1	1	1	1	8

### 3.2 Meta-analysis results

#### 3.2.1 Association between lncRNAs and LNM

**Fig. 2:**
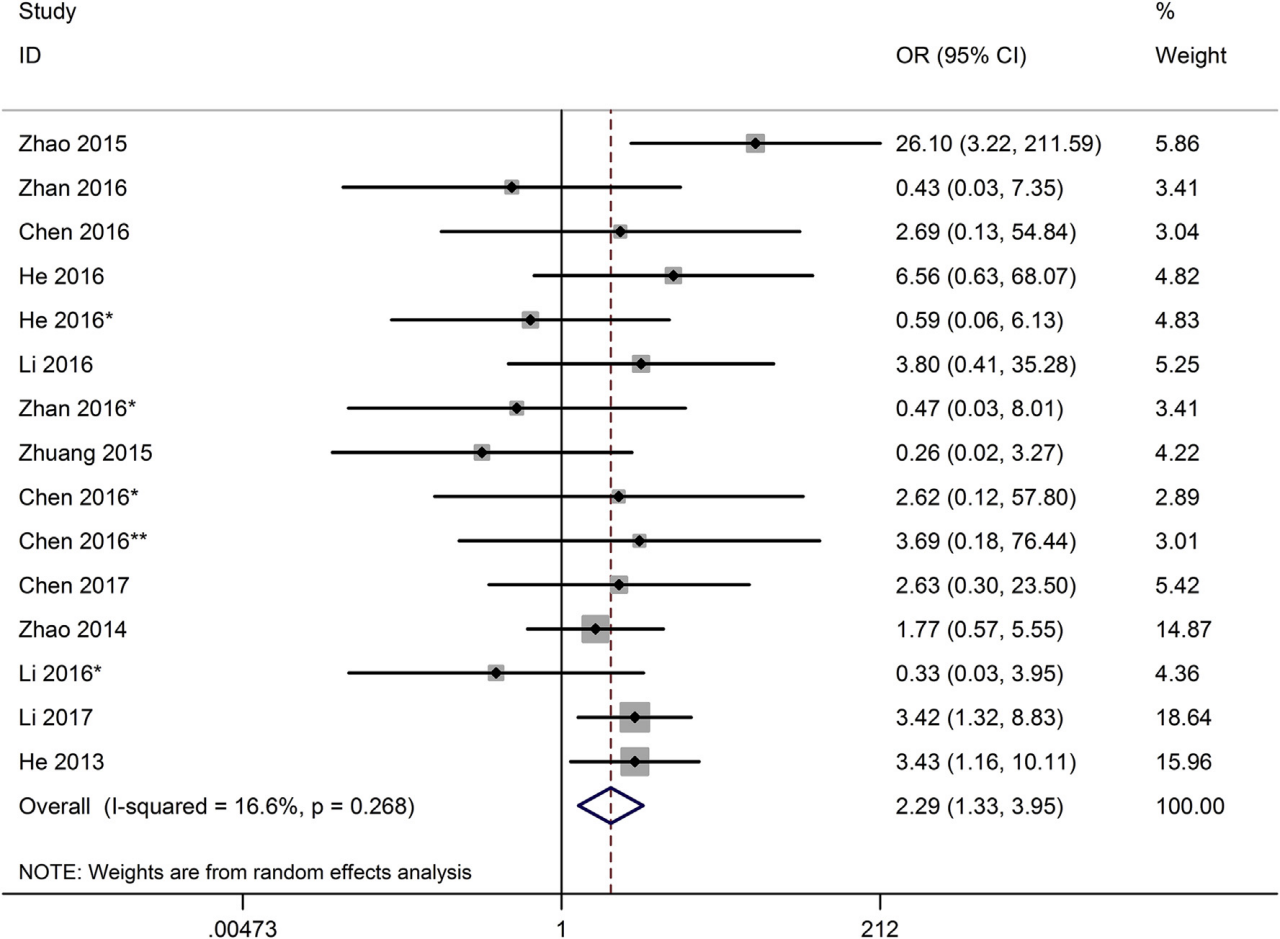
Forest plot for the association between lncRNAs expression with LNM.

One hundred and seventeen patients were included to assess the association between various kinds of lncRNAs expression level and LNM in bladder cancer. The random-effects model was expected to be adopted. Analysis showed the pooled OR was 2.29 (95 % CI 1.33–3.95, p < 0.01), which indicated that high expression of lncRNAs was predictive of LNM (Fig. 2). One of the studies showed that nearly half of the patients with high lncRNA-SPRY4-IT1 expression became LNM at last [33]. The result demonstrated that bladder cancer patients with high lncRNAs expression in tumor tissues were more susceptibility to develop LNM.

#### 3.2.2 Association between lncRNAs and T stage

**Fig. 3:**
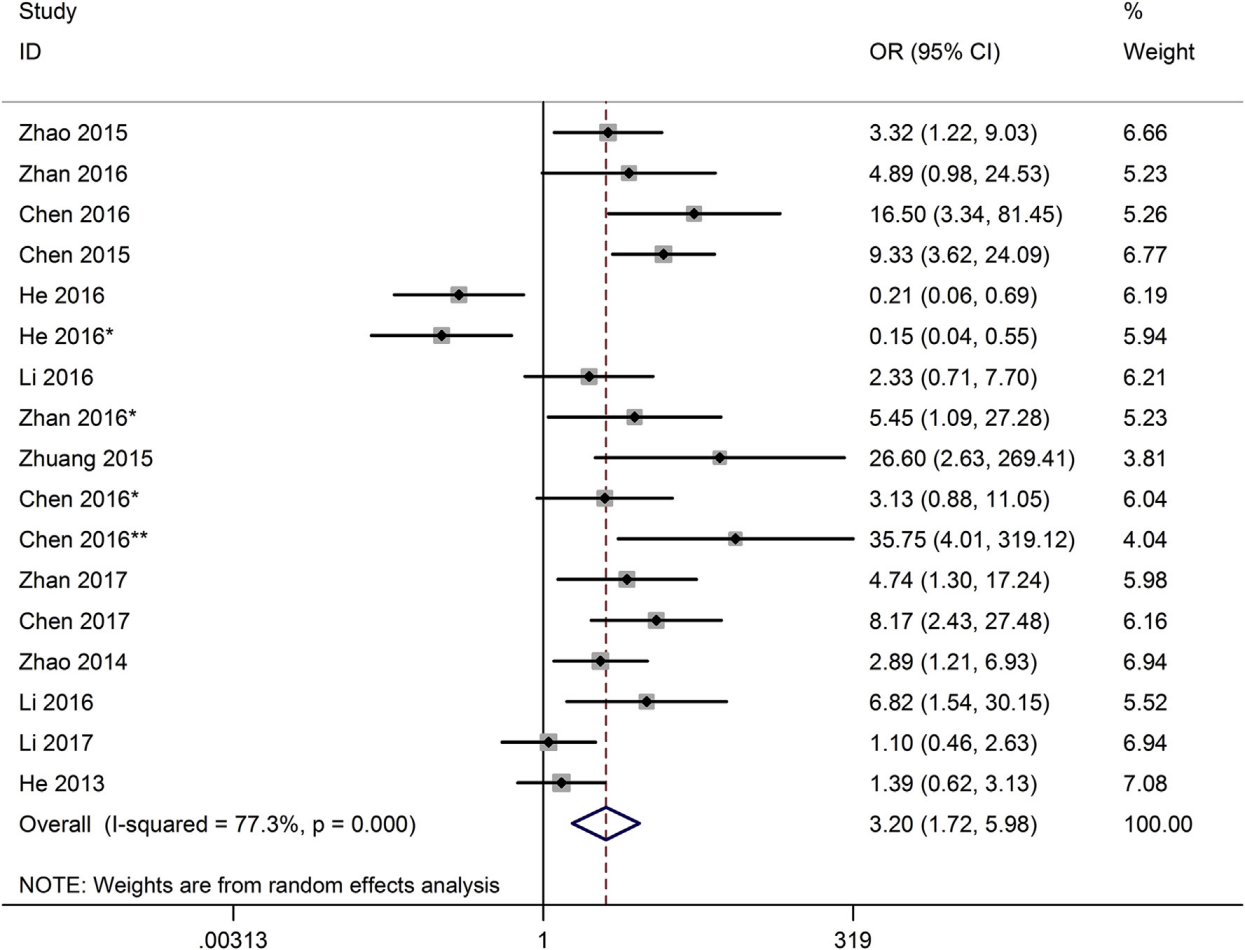
Forest plot for the association between lncRNAs expression with T stage (T).

Seventeen studies reporting a total of 1083 patients with T stage were included based on different lncRNAs expression patterns. According to T stage level (T1/2/3/4), we divided T1 into low T stage and T2-4 for high T stage group. The random-effects model was adopted. Analysis showed the OR of 3.20 with 95%CI: 1.72–5.98 (p < 0.001), which reveals that the expression of lncRNAs might be available predictors of high T stage (Fig. 3). In other words, in bladder cancer, high lncRNAs expression correlated with higher T stage. The results prove that the expression of lncRNAs in tumor tissues might be direct evidence of T stage.

#### 3.2.3 Association between lncRNAs and OS, RFS and PFS

**Fig. 4:**
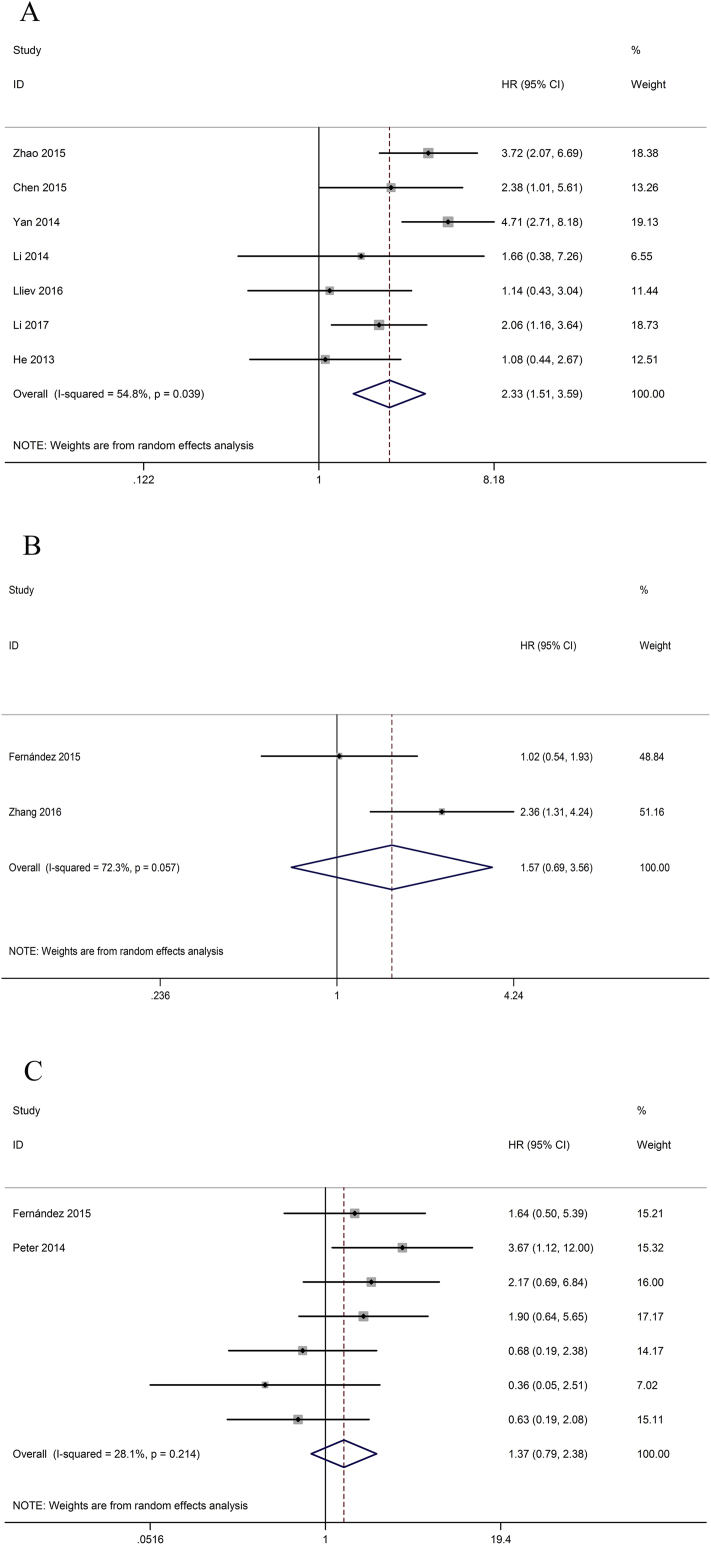
A. Forest plot of the correlation between lncRNAs expression levels and A. OS group; B. RFS group; and C. PFS group in different cancer patients.

We conducted the correlation between different LncRNAs expression level and OS among 622 patients diagnosed with bladder cancer from seven included studies. The relationship between LncRNAs expression level and OS of bladder cancer patients were found to be of significant heterogeneity (I2 = 54.8 %, p = 0.039), and the random model was applied. The pooled HR was 2.33 (95 % CI 1.51–3.59, p < 0.01), indicating that high lncRNAs expression level was associated with poorer OS of bladder cancer patients significantly (Fig. 4A). In other words, high lncRNAs expression correlated with a worse survival.

Two of the included studies reported the RFS of 181 patients according to lncRNAs expression levels. The random-effects model was used to calculate the pooled HR with corresponding 95% CI. According to meta-analysis result, it is known that high expression of lncRNAs might not be associated with poor RFS in tumors (pooled HR = 1.57, 95%CI 0.69–3.56, p = 0.284) (Fig. 4B). In a word, the cancer patients with high expression of lncRNAs might not be correlated with prognosis.

Two included studies reported a total of 155 patients with PFS according to lncRNAs expression levels. The random-effects model that was implemented to calculate the pooled HR with corresponding 95% CI. According to meta-analysis result (pooled HR = 1.37, 95%CI 0.79–2.38, p = 0.269) (Fig. 4C), it can be seen that the expression of lncRNAs might not be associated with poor PFS in bladder carcinoma. All the meta-analysis results were summarized in Table 3.

**Table 3:** Results of this meta-analysis.

Outcomes	No. of studies	No. of patients	HR/OR (95%CI)	P	Heterogeneity		Publication bias
					I^2^ (%)	Tau-square (%)	P-value
LNM	15	117	2.29 (1.33–3.95)	0.003	16.6	0.1808	0.268
T	17	1083	3.20 (1.72–5.98)	0.001	77.31	1.2619	0.001
OS	7	622	2.33 (1.51–3.59)	0.001	54.8	0.1743	0.039
RFS	2	181	1.57 (0.69–3.56)	0.284	72.3	0.2544	0.057
PFS	2	155	1.37 (0.79–2.38)	0.269	28.1	0.1555	0.214

LNM: lymph node metastasis; T: tumor state of cancers; OS: overall survival; RFS: recurrence free survival; PFS: progression free survival.

#### 3.2.4 Publication bias and sensitivity analysis

**Fig. 5:**
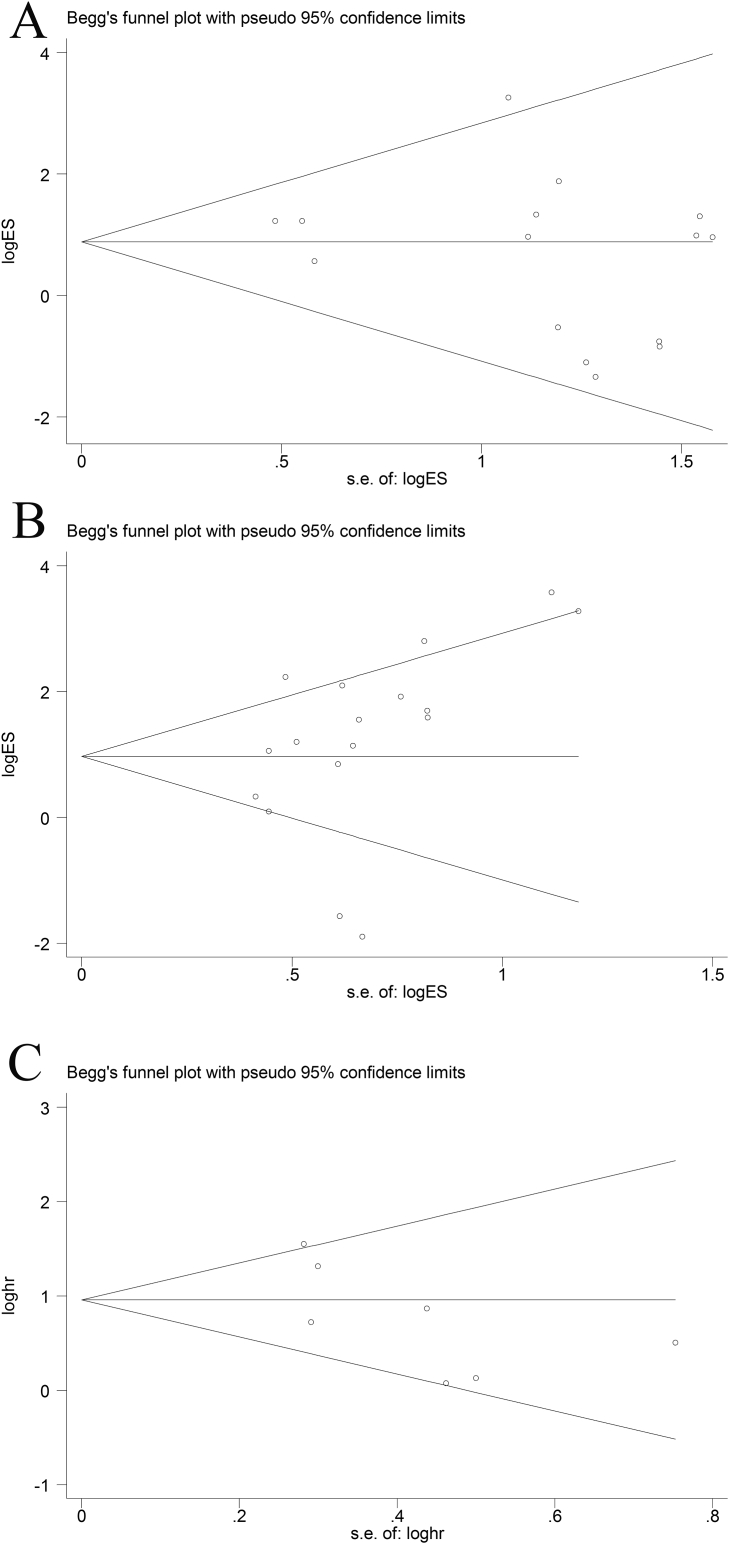
Funnel plot analysis of potential publication bias in A. LNM group; B. T stage group; and C. OS group (Egger's test).

**Fig. 6:**
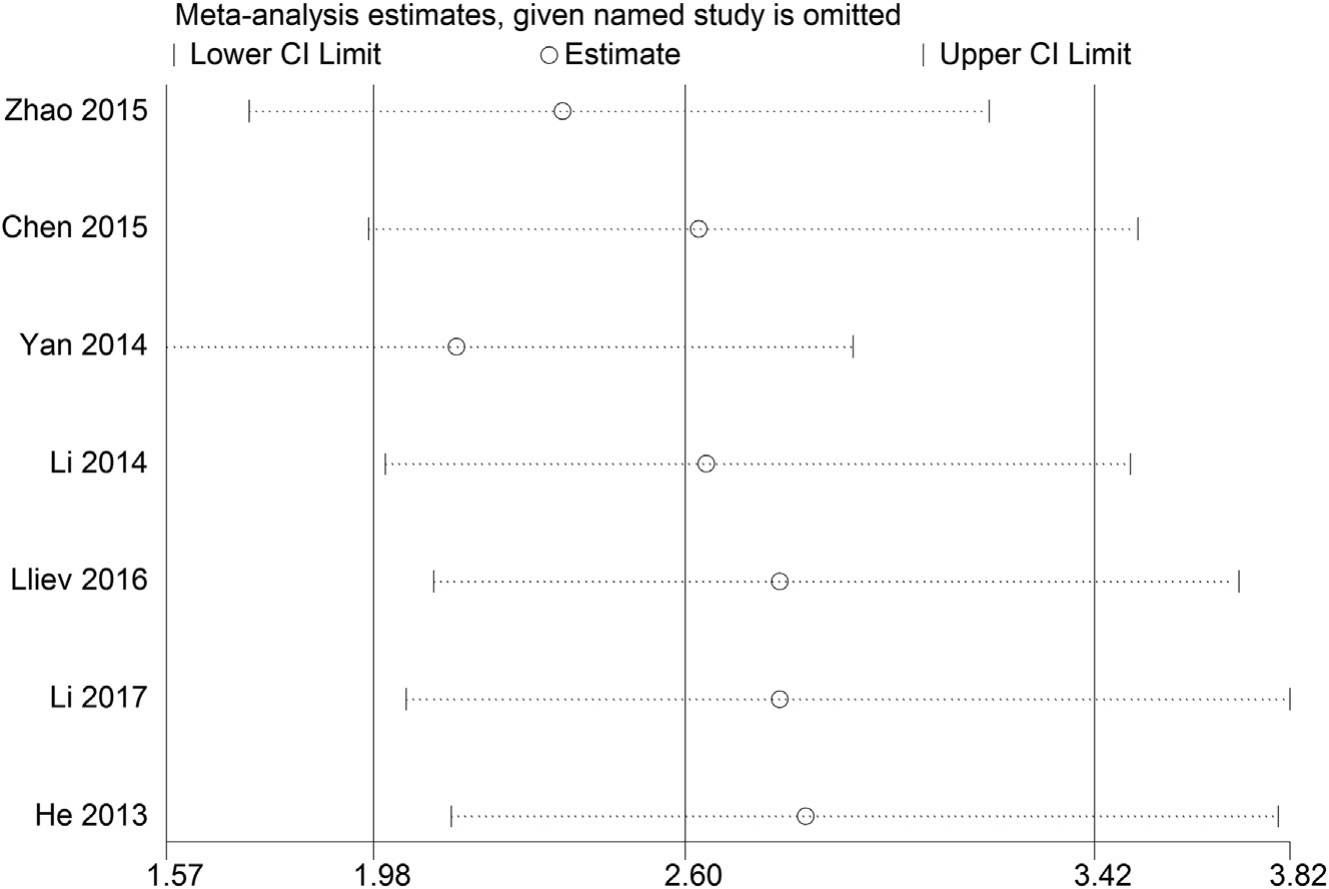
Sensitivity analysis of effect of individual studies on the pooled HRs for lncRNAs and overall survival of patients.

Publication bias of the present meta-analysis was evaluated by the Begg's funnel plot and Egger's test. In LNM group (Egger's test, t = -1.18, *p* = 0.258), T group (Egger's test, t = 1.59, *p* = 0.133) and OS group (Egger's test, t = -1.97, *p* = 0.106), the shapes of funnel plot were symmetric, no significant publication bias was observed by the Egger's test (Fig. 5). Sensitivity analysis is presented in Fig. 6. The result pattern was not significantly impacted by removing single study each time.

## 4 Discussion

The more we learned about lncRNAs, the more awareness we got that lncRNAs expression might predict poor OS in cancer patients. However, what methods should be taken to summarize the results of these experiments? In the clinic, meta-analysis is a commonly used research tool. Such analysis can summarize all the similar researchers and provide a direction in clinical work. However, the concept of combining meta-analysis is not easy; both statistical and biological analyzes are required. It is different from basic research for it is not a simple combination of all outcomes, but understanding and dealing of the intricate results with professional thinking, even sometimes the evidence is conflicting, it can improve our comprehension of biological systems.

This is the first meta-analysis to evaluate the association between multiple lncRNAs levels and clinical prognosis of bladder cancer. The present meta-analysis has been conducted to explore the correction between expression levels of lncRNAs and LNM, T stage, OS, RFS and PFS rate for bladder carcinoma patients. Our results shown in Table 3 demonstrated that the expression of lncRNAs in our retrieved research could predict poor survival in bladder cancer for patients. Through the above analysis, it can be seen that various lncRNAs might be a novel predictive factor of poor prognosis in bladder cancer patients. Meanwhile, these studies indicated that a signaling pathway can cause extracellular signaling molecules entering into the cell and can directly affect the phenotype of cells, such as cell proliferation, apoptosis, invasion, and metabolism. However, in this meta-analysis, we only focused on the function of these lncRNAs in bladder cancer, the mechanisms between them and the interrelationships are required in further experiments.

### 4.1 Limitations

Fairly, it should be recognized that the current meta-analysis still has some limitations. Firstly, statistical heterogeneity was detected in the studies. Heterogeneity may be caused by different types of lncRNA, clinical characteristics of patients, sample size, follow-up time and so on. Until now, it is still difficult to find a suitable way to deal with the issue of heterogeneity. What is more, the cut-off value and the method for detecting low or high levels of lncRNA varied in different studies, although there were conventional methods used to evaluate the expression of lncRNA, which may lead to heterogeneity of the results, and it was difficult to obtain a consensus cut-off value to define the overexpression in bladder cancer. Therefore, researchers need to develop a cut-off value with greater consistency, and to establish a method to classify high or low expression of lncRNA. Thirdly, we retrieved publications only written in English, and 24 studies with 1652 patients were included in the present meta-analysis eventually, so the total number of studies and patients included was relatively small. Importantly, in our analysis, the majority of the patients included in the article were Asian. The lack of diversity with respect to ancestry in lncRNA cancer studies was also a question that we cared about, which may determine whether the conclusion is universal or not. Hence, more and more future studies should be upheld for the results of this meta-analysis.

## 5 Conclusions

To sum up, despite the above limitations, the results of meta-analysis in this study could help us better understand the prognostic significance of different types of lncRNAs in bladder cancer. LncRNAs could be used as novel biomarkers for predicting the prognosis of bladder cancer and evaluating its clinical and pathological features. Ultimately, larger, multi-center, high-quality studies are needed for further scientific studies to validate the clinical application of lncRNAs in bladder cancer.

## Declarations

### Author contribution statement

Yucheng Zhong: Conceived and designed the experiments; Performed the experiments; Analyzed and interpreted the data; Contributed reagents, materials, analysis tools or data; Wrote the paper.

Yeshen Zhang: Conceived and designed the experiments; Performed the experiments; Contributed reagents, materials, analysis tools or data.

Huan Li: Conceived and designed the experiments; Performed the experiments; Analyzed and interpreted the data; Contributed reagents, materials, analysis tools or data.

Wenmin Ma: Conceived and designed the experiments; Analyzed and interpreted the data; Contributed reagents, materials, analysis tools or data; Wrote the paper.

### Funding statement

This research did not receive any specific grant from funding agencies in the public, commercial, or not-for-profit sectors.

### Competing interest statement

The authors declare no conflict of interest.

### Additional information

No additional information is available for this paper.

## References

[1] Chen W., Zheng R., Baade P.D., Zhang S., Zeng H., Bray F., Jemal A., Yu X.Q., He J. (2016). Cancer statistics in China, 2015. CA Cancer J. Clin..

[2] Resnick M.J. (2016). Variation in bladder cancer spending: a global call to action. Eur. Urol..

[3] Antoni S., Ferlay J., Soerjomataram I., Znaor A., Jemal A., Bray F. (2017). Bladder cancer incidence and mortality: a global overview and recent trends. Eur. Urol..

[4] Rentsch C.A., Müller D.C., Ruiz C., Bubendorf L. (2017). Comprehensive molecular characterization of urothelial bladder carcinoma: a step closer to clinical translation?. Eur. Urol..

[5] Jin X., Yun S.J., Jeong P., Kim I.Y., Kim W.J., Park S. (2014). Diagnosis of bladder cancer and prediction of survival by urinary metabolomics. Oncotarget.

[6] Necsulea A., Soumillon M., Warnefors M., Liechti A., Daish T., Zeller U., Baker J.C., Grützner F., Kaessmann H. (2014). The evolution of lncRNA repertoires and expression patterns in tetrapods. Nature.

[7] Gibb E.A., Brown C.J., Lam W.L. (2011). The functional role of long non-coding RNA in human carcinomas. Mol. Cancer.

[8] Cheng L., Davison D.D., Adams J., Lopez-Beltran A., Wang L., Montironi R., Zhang S. (2014). Biomarkers in bladder cancer: translational and clinical implications. Crit. Rev. Oncol. Hematol..

[9] Liberati A., Altman D.G., Tetzlaff J., Mulrow C., Gøtzsche P.C., Ioannidis J.P., Clarke M., Devereaux P.J., Kleijnen J., Moher D. (2009). The PRISMA statement for reporting systematic reviews and meta-analyses of studies that evaluate healthcare interventions: explanation and elaboration. BMJ.

[10] Zhao X.L., Zhao Z.H., Xu W.C., Hou J.Q., Du X.Y. (2015). Increased expression of SPRY4-IT1 predicts poor prognosis and promotes tumor growth and metastasis in bladder cancer. Int. J. Clin. Exp. Pathol..

[11] Zhan Y., Liu Y., Wang C., Lin J., Chen M., Chen X., Zhuang C., Liu L., Xu W., Zhou Q., Sun X., Zhang Q., Zhao G., Huang W. (2016). Increased expression of SUMO1P3 predicts poor prognosis and promotes tumor growth and metastasis in bladder cancer. Oncotarget.

[12] Chen T., Xie W., Xie L., Sun Y., Zhang Y., Shen Z., Sha N., Xu H., Wu Z., Hu H., Wu C. (2015). Expression of long noncoding RNA lncRNA-n336928 is correlated with tumor stage and grade and overall survival in bladder cancer. Biochem. Biophys. Res. Commun..

[13] He A., Chen Z., Mei H., Liu Y. (2016). Decreased expression of LncRNA MIR31HG in human bladder cancer. Cancer Biomark..

[14] He A., Liu Y., Chen Z., Li J., Chen M., Liu L., Liao X., Lv Z., Zhan Y., Zhuang C., Lin J., Huang W., Mei H. (2016). Over-expression of long noncoding RNA BANCR inhibits malignant phenotypes of human bladder cancer. J. Exp. Clin. Cancer Res..

[15] Li J., Zhuang C., Liu Y., Chen M., Chen Y., Chen Z., He A., Lin J., Zhan Y., Liu L., Xu W., Zhao G., Guo Y., Wu H., Cai Z., Huang W. (2016). Synthetic tetracycline-controllable shRNA targeting long non-coding RNA HOXD-AS1 inhibits the progression of bladder cancer. J. Exp. Clin. Cancer Res..

[16] Martínez-Fernández M., Feber A., Dueñas M., Segovia C., Rubio C., Fernandez M., Villacampa F., Duarte J., López-Calderón F.F., Gómez-Rodriguez M.J., Castellano D., Rodriguez-Peralto J.L., de la Rosa F., Beck S., Paramio J.M. (2015). Analysis of the Polycomb-related lncRNAs HOTAIR and ANRIL in bladder cancer. Clin. Epigenet..

[17] Yan T.H., Lu S.W., Huang Y.Q., Que G.B., Chen J.H., Chen Y.P., Zhang H.B., Liang X.L., Jiang J.H. (2014). Upregulation of the long noncoding RNA HOTAIR predicts recurrence in stage Ta/T1 bladder cancer. Tumour Biol..

[18] Zhan Y., Lin J., Liu Y., Chen M., Chen X., Zhuang C., Liu L., Xu W., Chen Z., He A., Zhang Q., Sun X., Zhao G., Huang W. (2016). Up-regulation of long non-coding RNA PANDAR is associated with poor prognosis and promotes tumorigenesis in bladder cancer. J. Exp. Clin. Cancer Res..

[19] Zhang S., Zhong G., He W., Yu H., Huang J., Lin T. (2016). lncRNA up-regulated in nonmuscle invasive bladder cancer facilitates tumor growth and acts as a negative prognostic factor of recurrence. J. Urol..

[20] Zhuang C., Li J., Liu Y., Chen M., Yuan J., Fu X., Zhan Y., Liu L., Lin J., Zhou Q., Xu W., Zhao G., Cai Z., Huang W. (2015). Tetracycline-inducible shRNA targeting long non-coding RNA PVT1 inhibits cell growth and induces apoptosis in bladder cancer cells. Oncotarget.

[21] Chen Z., Liu Y., He A., Li J., Chen M., Zhan Y., Lin J., Zhuang C., Liu L., Zhao G., Huang W., Cai Z. (2016). Theophylline controllable RNAi-based genetic switches regulate expression of lncRNA TINCR and malignant phenotypes in bladder cancer cells. Sci. Rep..

[22] Chen M., Zhuang C., Liu Y., Li J., Dai F., Xia M., Zhan Y., Lin J., Chen Z., He A., Xu W., Zhao G., Guo Y., Cai Z., Huang W. (2016). Tetracycline-inducible shRNA targeting antisense long non-coding RNA HIF1A-AS2 represses the malignant phenotypes of bladder cancer. Cancer Lett..

[23] Peter S., Borkowska E., Drayton R.M., Rakhit C.P., Noon A., Chen W., Catto J.W. (2014). Identification of differentially expressed long noncoding RNAs in bladder cancer. Clin. Cancer Res..

[24] Zhan Y., Li Y., Guan B., Chen X., Chen Z., He A., He S., Gong Y., Peng D., Liu Y., Cai Z., Li X., Zhou L. (2017). Increased expression of long non-coding RNA CCEPR is associated with poor prognosis and promotes tumorigenesis in urothelial bladder carcinoma. Oncotarget.

[25] Chen M., Li J., Zhuang C., Cai Z. (2017). Increased lncRNA ABHD11-AS1 represses the malignant phenotypes of bladder cancer. Oncotarget.

[26] Zhao F., Lin T., He W., Han J., Zhu D., Hu K., Li W., Zheng Z., Huang J., Xie W. (2015). Knockdown of a novel lincRNA AATBC suppresses proliferation and induces apoptosis in bladder cancer. Oncotarget.

[27] Li L.J., Zhu J.L., Bao W.S., Chen D.K., Huang W.W., Weng Z.L. (2014). Long noncoding RNA GHET1 promotes the development of bladder cancer. Int. J. Clin. Exp. Pathol..

[28] Li J., Zhuang C., Liu Y., Chen M., Zhou Q., Chen Z., He A., Zhao G., Guo Y., Wu H., Cai Z., Huang W. (2016). shRNA targeting long non-coding RNA CCAT2 controlled by tetracycline-inducible system inhibits progression of bladder cancer cells. Oncotarget.

[29] Iliev R., Kleinova R., Juracek J., Dolezel J., Ozanova Z., Fedorko M., Pacik D., Svoboda M., Stanik M., Slaby O. (2016). Overexpression of long non-coding RNA TUG1 predicts poor prognosis and promotes cancer cell proliferation and migration in high-grade muscle-invasive bladder cancer. Tumour Biol..

[30] Li C., Cui Y., Liu L.F., Ren W.B., Li Q.Q., Zhou X., Li Y.L., Li Y., Bai X.Y., Zu X.B. (2017). High expression of long noncoding RNA MALAT1 indicates a poor prognosis and promotes clinical progression and metastasis in bladder cancer. Clin. Genitourin. Cancer.

[31] Zhang H., Guo Y., Song Y., Shang C. (2017). Long noncoding RNA GAS5 inhibits malignant proliferation and chemotherapy resistance to doxorubicin in bladder transitional cell carcinoma. Cancer Chemother. Pharmacol..

[32] He W., Cai Q., Sun F., Zhong G., Wang P., Liu H., Luo J., Yu H., Huang J., Lin T. (2013). linc-UBC1 physically associates with polycomb repressive complex 2 (PRC2) and acts as a negative prognostic factor for lymph node metastasis and survival in bladder cancer. Biochim. Biophys. Acta.

[33] Li J., Li Z., Leng K., Xu Y., Ji D., Huang L., Cui Y., Jiang X. (2018). ZEB1-AS1: a crucial cancer-related long non-coding RNA. Cell Prolif.

